# Chromosome Level Genome Assembly of *Andrographis paniculata*

**DOI:** 10.3389/fgene.2020.00701

**Published:** 2020-06-30

**Authors:** Ying Liang, Shanshan Chen, Kunhua Wei, Zijiang Yang, Shengchang Duan, Yuan Du, Peng Qu, Jianhua Miao, Wei Chen, Yang Dong

**Affiliations:** ^1^Guangxi Key Laboratory of Medicinal Resources Protection and Genetic Improvement, Guangxi Botanical Garden of Medicinal Plants, Nanning, China; ^2^BGI College, Zhengzhou University, Zhengzhou, China; ^3^National and Local Joint Engineering Research Center on Germplasm Innovation and Utilization of Chinese Medicinal Materials in Southwest China, Yunnan Agricultural University, Kunming, China; ^4^NowBio Biotechnology Company, Kunming, China; ^5^State Key Laboratory for Conservation and Utilization of Bio-Resources in Yunnan, Yunnan Agricultural University, Kunming, China; ^6^College of Agronomy and Biotechnology, Yunnan Agricultural University, Kunming, China; ^7^Yunnan Research Institute for Local Plateau Agriculture and Industry, Kunming, China

**Keywords:** PacBio sequencing, Hi-C, genome assembly, medicinal plant, *Andrographis paniculata*

## Abstract

*Andrographis paniculata* (Chinese name: Chuanxinlian) is an annual dicotyledonous medicinal plant widely grown in China and Southeast Asia. The dried plant has a highly acclaimed usage in the traditional Chinese medicine for its antipyretic, anti-inflammatory, and analgesic effects. In order to help delineate the biosynthetic pathways of various secondary metabolites, we report in this study a high-quality reference genome for *A. paniculata*. With the help of both PacBio single molecule real time sequencing and Illumina sequencing reads for error correction, the *A. paniculata* genome was assembled into a total size of 284 Mb with a contig N50 size of 5.14 Mb. The contigs were further assembled into 24 pseudo-chromosomes by the Hi-C technique. We also analyzed the gene families (e.g., *KSL*, and *CYP450*) whose protein products are essential for synthesizing bioactive compounds in *A. paniculata.* In conclusion, the high-quality *A. paniculata* genome assembly builds the foundation for decoding the biosynthetic pathways of various medicinal compounds.

## Introduction

During the course of human history, all civilizations have tried to explore various plants for medicinal purposes and they formed unique empirical knowledge about how these herbal plants could be used to treat various diseases. Even though much of this knowledge has gradually given way to modern medicine, WHO found that the popularity of herbal medicines increased in almost all parts of the world in recent years (Fitzsimons, 2013). For this reason, herbal plants not only raise the enthusiasm from the general public, but also remain a rich source for discovering novel drug candidates among the researchers.

With the emergence and development in high-throughput sequencing technology, the genome sequences of more than 200 plants have been reported (Lin et al., 2011). Particularly, genome sequencing is a powerful tool for studying various aspects of physiology and genetics in non-model plants, many of which are traditional herbal plants (Zerikly and Challis, 2009; De Luca et al., 2012; Chae et al., 2014). For example, the reference genome of *Scutellaria baicalensis* (Zerikly and Challis, 2009; De Luca et al., 2012; Chae et al., 2014; Zhao et al., 2019), *Panax ginseng* (Xu et al., 2017), mint (Vining et al., 2017), and opium poppy (Guo et al., 2018) provided insights into the genes involved in the biosynthesis of unique flavonoids, terpenes, alkaloids and many other secondary metabolites. Additionally, the reference genome of *Salvia splendens* was very valuable for helping marker-assisted breeding, genome editing, and molecular genetics (Dong et al., 2018). As one of the participants of the Herbal Plant Genomics Initiative, our team has reported the genomes of many Chinese herbal plants in past years, including *Salvia miltiorrhiza Bunge* (Zhang et al., 2015), *Dendrobium officinale* (Yan et al., 2015), maca (Zhang et al., 2016), *Panax notoginseng* (Chen et al., 2017), and fleabane (Yang et al., 2017). The high-quality genome assembly of *Andrographis paniculata* is presented in this manuscript as a continuum of the bigger research project.

**FIGURE 1:**
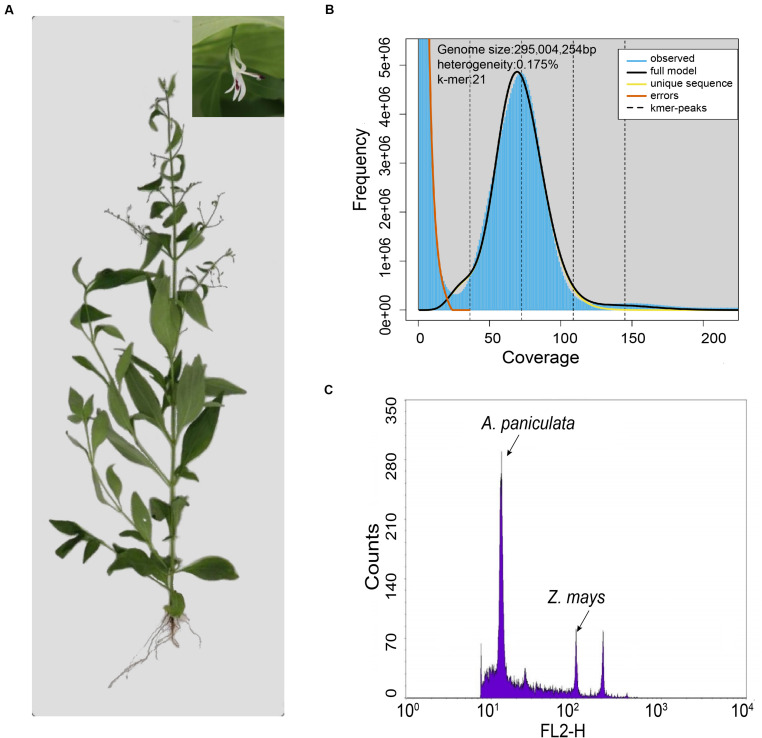
Evaluation of the genome size of *A. paniculata*. **(A)** Photo of a single *A. paniculata* plant with close-up image of the flower. **(B)** The 21*-mer* analysis of the *A. paniculata* genome using GenomeScope. **(C)** Flow cytometry analysis of *A. paniculata* genome size comparing with *Zea mays*.

*Andrographis paniculata* (Figure 1A) is a dicotyledonous medicinal plant widely distributed and used in tropical and subtropical regions of Asia, including India, China, Thailand, and Malaysia (Lim et al., 2012). This annual plant belongs to the family of Acanthaceae in the order of Lamiales. The dried plant has a highly acclaimed usage in the traditional Chinese medicine for its antipyretic, anti-inflammatory, and analgesic effects (Sun et al., 2019). Previous pharmacological research identified andrographolide and neoandrographolide as the main therapeutic constituents in *A. paniculata* (Srivastava and Akhila, 2010). Andrographolide is a labdane-related diterpenoid and it exhibits anti-cancer (Luo et al., 2014), anti-virus (Chen et al., 2009), antimicrobial and anti-inflammatory activities (Chua, 2014), suggesting potential pharmaceutical values. The leaves of *A. paniculata* contain major amounts of diterpene lactone compounds, including about 0.1% of deoxyandrographolide, and about 0.2% of neoandrographolide (Srivastava and Akhila, 2010). Even though the biosynthesis of andrographolide and neoandrographolide is achieved by the combination of various isopentenyl diphosphate (IPP) and dimethylallyl diphosphate (DMAPP) (Chen et al., 2011), the complete profile of *CYTOCHROME P450* genes (*CYPs*), *COPALYL DIPHOSPHATE SYNTHASE* genes (*CPSs*), and *KAURENE SYNTHASE-LIKE PROTEIN* genes (*KSLs*) has not been fully investigated in the *A. paniculata* genome.

The genome of *A. paniculata* is highly heterozygous and contains many repetitive sequences. These characteristics pose a big challenge in terms of acquiring a high-quality whole-genome reference assembly. A previous research effort reported an *A. paniculata* genome assembly of ∼269 Mb in total size with a contig N50 of 388 Kb (Sun et al., 2019). These benchmarks suggest the existence of relatively large un-assembled genome and gaps among contigs. Therefore, it is valuable to improve the genome assembly of *A. paniculata* using better raw data and assembly pipeline. Herein, we reported a reference genome of *A. paniculata* obtained from the PacBio single-molecule real time sequencing data in the size of 284 Mb. The contig N50 size was improved to 5.14 Mb, which is more than 12-fold longer than before. The resultant contigs were further assembled into 24 pseudo-chromosomes by the Hi-C technology, thereby yielding a high-quality *A. paniculata* genome assembly.

## Materials and Methods

### DNA Extraction, Library Construction, and Sequencing

A single *A. paniculata* individual was obtained from Guangxi Medicinal Botanical Garden. Plant DNA was extracted from young leaves with the Novel Plant Genomic DNA Rapid Extraction Kit (Genenode Biotech, Beijing, China) according to the product manual.

A total of 15 μg *A. paniculata* genomic DNA was used to construct eleven PacBio libraries (mean size of 20 kb) with the SMRT Template Prep Kit (Pacific Biosciences, United States). These libraries were sequenced on a PacBio Sequel platform with recommended protocols from the manufacturer (Supplementary Table 1). Sequence reads with a quality score lower than 0.8 were removed.

In order to perform genome survey and genomic base correction, we also obtained Illumina reads for *A. paniculata.* In brief, 2 μg of genomic DNA was used to construct each library. The genomic DNA was sheared and insert sizes of 241, 313, 424, and 533 bp were selected for the four libraries. All libraries were sequenced on an Illumina HiSeq X Ten platform. The Illumina raw data were processed by fq_filter_V1.5 to remove the low-quality reads, and the parameters were set as follows: -q 33 -t 20 -ta 5 -tb 10 -tc 5 -td 10. We filtered out low-quality reads as specified by the following criteria: (1) filter a read if more than 5% of bases were N or poly-A, (2) filter a read if more than 30 bases were low quality, (3) if the read was contaminated with adaptor sequence, (4) if the size of a read was too small, and (5) if two copies of the paired-end reads had identical sequence (remove both copies). The resultant reads were then corrected by the SOAPec_v2.0.1 package with default settings.

### Genome Size Estimation

The genome size of *A. paniculata* was assessed by both flow cytometry and *k*-mer analysis. For the flow cytometry approach, 20 mg of plant tissue was placed in 1.0 ml ice-cold nuclei isolation buffer in a Petri dish. The plant tissue was minced in the buffer with a new razor blade. The homogenate was filtered through a 42-mm nylon mesh into a labeled sample tube. Propidium iodide was added to a final concentration of 50 mg/ml simultaneously with RNase (50 mg/ml) and the sample was incubated on ice for 20 min before measurement. *Zea mays* (B73) was used as internal standard, the internal reference maize genome size is 2.3 Gb (Schnable et al., 2009).

For the *k-mer* analysis, Jellyfish v.2.2.5 (Marcais and Kingsford, 2011) was used to perform *k-mer* analysis on the Illumina sequencing error-corrected data. First, FastQC software was used to identify the quality of the input sequencing data for the Illumina sequencing data. The first 500,000,000 lines of the lane2 fastq file were extracted, and the extracted reads were used to identify heterozygosity with the ‘–m 21’ option. The graph was generated by GenomeScope (Vurture et al., 2017).

### Genome Assembly

A total of 53.98 Gb of PacBio data were used in the *de novo* assembly of the *A. paniculata* genome according to an assembly pipeline named HERA (Du and Liang, 2019). In brief, CANU v1.8 (Koren et al., 2017) was used to correct the raw PacBio data and assemble contigs. The resultant contigs were further improved with HERA. Next, the pipeline used the assembled genome to create an index file using bwa-mem (Li and Durbin, 2010). The processed next-generation sequencing data were aligned to the reference genome. Samtools (Li et al., 2009) was used to sort the resulting bam files. Finally, the bam files were used to polish the contig twice with Pilon (Walker et al., 2014). In this study, a commercial service provider from the Institute of Genetics and Developmental Biology in Beijing was recruited to assemble the *A. paniculata* genome using a professional version of HERA (Du and Liang, 2019), which is said to run faster and have better performance resolving repetitive sequences.

### Hi-C Library Construction and Pseudomolecule Clustering

Three gram of *A. paniculata* leaves were harvested and crosslinked in a 2% formaldehyde solution for 15min at room temperature. Crosslinking was quenched by adding glycine to a final concentration of 250 mM. The fixed plant tissue was then ground in liquid nitrogen and suspended in extraction buffer for nuclei isolation. After the nuclei were separated, chromatin was solubilized in 0.1% (m/v) SDS at 65°C for 10 min. After SDS was quenched by Triton X-100 (final concentration of 1%), solubilized chromatin was digested by 400 units of *Dpn*II (New England Biolabs, MA, United States) at 37°C overnight. The following steps included biotin labeling of the DNA and blunt-end ligation of DNA fragments. After cross-linking was reversed by the treatment with proteinase K, DNA was purified so that biotin labels could be removed from non-ligated fragment ends. DNA fragments were sonicated into sizes of 400 bp so that paired-end libraries could be obtained. These libraries were sequenced on a NovaSeq 6000 platform (Illumina, United States) to acquire the Hi-C data.

Low-quality Hi-C reads were removed according to the following two criteria: (1) filter a read if more than 10% of bases were N, (2) filter a read if more than 50% bases were low quality (*Q* ≤ 5). Clean Hi-C reads were mapped to the draft assembly with Juicer (Juicer, juicer_tools.1.7.6_jcuda.0.8.jar) (Durand et al., 2016a). A candidate chromosome-length assembly was generated automatically using the 3d-DNA pipeline to correct misjoins, order, orientation, and then anchor contigs from the draft assembly (Dudchenko et al., 2017). Manual review and refinement of the candidate assembly was performed in Juicebox Assembly Tools (Version 1.9.1) (Durand et al., 2016b) for quality control and interactive correction. And then the genome was finalized using the “run-asm-pipeline-post-review.sh -s finalize –sort-output –bulid-gapped-map” in 3d-DNA with manually adjusted assembly as input (Dudchenko et al., 2017).

### Genome Annotation

The repetitive sequences were identified via sequence alignment and *de novo* prediction. RepeatMasker (Chen, 2004) was used to compare the assembled genome with the RepBase database (Release16.10)^1^ using default settings (Bao et al., 2015). Repeatproteinmask searches^2^ were used for prediction of homologs using default settings. For *de novo* annotation of repetitive elements, LTR_finder (Xu and Wang, 2007)^3^, Piler (Edgar and Myers, 2005)^4^, RepeatScout (Price et al., 2005)^5^, and RepeatModeler^6^ were used to construct the *de novo* library, and annotation was carried out with Repeatmasker (cutf 100, cpu 100, run qsub, -nolow,-no, -norna,-parallel 1). Tandem repeats were identified across the genome with Tandem Repeats Finder (cutf 100, cpu 100, period_size 2000, run qsub Match 2 Mismatch 7 Delta 7 PM 80 PI 10 Minscore 50 MaxPeriod 2000).

According to their characteristics and redundancy, the repeat consensus sequences were first classified using TEsort (Zhang et al., 2019) with REXdb database^7^. For *Copia* and *Gyspy* superfamilies, complete elements were identified based on the presence and order of conserved domains including capsid protein, aspartic proteinase, integrase, reverse transcriptase and RNase H as described in Wicker (Wicker and Keller, 2007). We extracted all reverse transcriptase and multiple sequence alignment of the extracted RT were then conducted by MAFFT (Nakamura et al., 2018) and the phylogenetic tree was constructed with IQTREE (Jana et al., 2016). Itol^8^ was used for the visualization and edit of the tree. Finally, density of the TE consensus copies according to their lineages were computed along pseudomolecules and visualized using R.

GlimmerHMM (Aggarwal and Ramaswamy, 2002), SNAP (Korf, 2004), GenScan (Aggarwal and Ramaswamy, 2002), and Augustus (Stanke et al., 2006) were used for *ab initio* prediction of protein-coding genes with default settings. The homology-based prediction utilized reference protein sequence from *Arabidopsis thaliana* (Michael et al., 2018), *Sesamum indicum* (Wang et al., 2014), *Solanum lycopersicum* (Consortium, 2012), and *Vitis vinifera* (Girollet et al., 2019) according to an established protocol. RNA-seq data sets for *A. paniculata* leaf and root tissues were obtained from the National Center for Biotechnology Information (NCBI) database (SRX652837, SRX655521), and subsequently used for *de novo* assembly of the transcriptome. We aligned all RNA reads to the *A. paniculata* genome using TopHat (Trapnell et al., 2009), assembled the transcripts with Cufflinks (Trapnell et al., 2013) using default parameters, and predicted the open reading frames to obtain reliable transcripts with hidden Markov model (HMM)-based training parameters. Finally, the above 3 gene structure models were compiled by Evidence Modeler tool (Haas et al., 2008) with the following weights: transcripts-set > homology-set > *ab initio*-set and redundant genes were removed.

The t-RNAscan-SE tool (Chan and Lowe, 2019) was used to predict tRNA in the genome sequence with *E*-value set to 1e-5. Plant RNA sequences from Rfam database were selected as reference to predict the rRNA by BLASTN with E-value set to 1e-5. The miRNA and snRNA genes were also predicted by BLASTN against the Rfam database with *E*-value set to e-1.

Gene function was annotated by performing BLASTP (*E*-value ≤ 1e-5) against the protein databases. SwissProt^9^, TrEMBL (see footnote 9), KEGG^10^, and InterPro^11^ were used for screening the functional domains of the proteins. Gene Ontology (GO) terms for each gene were extracted from the corresponding InterPro entries.

### Evolutionary and Phylogenetic Analyses

Protein sequences of *A. paniculata*, *Sesamum indicum*, *Salvia miltiorrhiza*, *Oryza sativa*, *Catharanthus roseus, Arabidopsis thaliana, Solanum lycopersicum*, and *Helianthus annuus* were downloaded from JGI. A full alignment protein search using BLASTP with the parameter *E*-value = 1e-5 was performed to verify the gene family clusters in these species and *A. paniculata*. Ortholog clustering and gene family clustering analyses were performed using OrthoMCL (Li et al., 2003). Venn diagram format was drawn using a web tool^12^ (Zhang et al., 2016).

An all-against-all BLASTP comparison with a cutoff *E*-value of 1e-5 was preformed, and the results were clustered into groups of homologous proteins using Markov chain clustering with the default inflation parameter. All 1212 single-copy orthologous genes identified in the gene family cluster analysis from the aforementioned species were used to construct a phylogenetic tree. Multiple sequence alignments were performed for each gene using MUSCLE v.3.7^13^ with default settings (Edgar, 2004).

The MCMCTREE program within the PAML package (Yang, 2007) was used to estimate divergence time of *A. paniculata*, *S. indicum*, *S. miltiorrhiza*, *O. sativa*, *C. s roseus, A. thaliana, S. lycopersicum*, and *H. annuus*. The HKY85 model (model = 4) and independent rates molecular clock (clock = 2) were used for calculation.

CAFE v1.7 (De Bie et al., 2006) is a tool for analyzing the evolution of gene family size based on the stochastic birth and death model. With the calculated phylogeny and the divergence time, this software was applied to identify gene families that had undergone expansion and/or contraction in the aforementioned species with the parameters: -filter -cpu 10 -lrt -simunum 1000.

### Synteny Analysis of Two Genome Assemblies

The fasta and hic.gff files of the published genome assembly (Sun et al., 2019) were downloaded from NCBI. They were combined with the fasta and contig.gff files of our genome assembly by makeblastdb. BLASTP was used to align these sequence, and MCScanX (Wang et al., 2012) was used to perform synteny analysis between two genome assemblies.

### Analysis of Key Gene Families in the *A. paniculata* Genome

We used hmmsearch to perform a preliminary screening of the gene family (*CYP450* and *terpene sythases, TPSs*) in *A. paniculata* and the gene ID was intercepted with an *E*-value ≤ 1e-59. The corresponding protein sequence was used as a query for TBLASTN (*E* = 1e-5) with both versions of the assembled *A. paniculata* genome sequence. The *CYP450* genes from other species were downloaded from the Cytochrome *CYP450* homepage^14^ (Nelson, 2009) and the *TPS* genes from other species were acquired from a previous publication (Chen et al., 2011). Multiple sequence alignment was carried out with MUSCLE v3.7 (Edgar, 2004) using default parameters. The maximum likelihood (ML) phylogenetic tree was constructed using MEGA7 (Nelson, 2009) with 1,000 bootstraps.

The RNA-Seq data of the roots and leaves of *A. paniculata* were downloaded from the NCBI database (SRX652837, SRX655521). FPKM value was calculated for each protein-coding gene by Cufflinks (v. 2.1.1) (Trapnell et al., 2013). The heatmap was made with the pheatmap package.

## Results

### Genomic Sequencing and High-Quality Genome Assembly

Genome survey with Illumina reads showed that the estimated genome size of *A. paniculata* with *21-mer* analysis was about 295 Mb (Figure 1B). This number was slightly smaller than the estimated genome size of 310 Mb from flow cytometry analysis (Figure 1C), but larger than the previous estimate of 280 Mb (Sun et al., 2019). The difference probably reflects the between-individual variation of the *A. paniculata* plant. Moreover, the heterozygosity of this sequenced genome was estimated to be 0.175% (Figure 1B).

In order to obtain a high-quality *A. paniculata* genome assembly, we constructed 11 PacBio SMRT sequencing libraries, which produced 53.96 Gb clean data (Supplementary Table 1). They covered about 183-fold of the estimated genome. We also generated about 238.6 Gb of Illumina sequencing data to polish and correct the error reads that are associated with PacBio sequencing. A combination of these data through a assembly pipeline (see the section on genome assembly methods for details) yielded a draft genome assembly of ∼284.3 Mb with 270 contigs (Table 1). This assembly represented about 91.6 – 96.3% of the estimated genome size. The longest contig was 9.30 Mb in size and the contig N50 was about 5.15 Mb. This benchmark is more than 12-fold longer than that of a previously reported assembly (Sun et al., 2019).

**TABLE 1:** The library information and data statistics for the *A. paniculata* assembly.

**Estimated genome size (Mb)**	**295 - 310**
**Assembly statistics**	
Assembly size (Mb) Number of N50 contig	284.3 22
N50 contig length (bp)	5,149,272
Number of N90 contigs	51
N90 contig length (bp)	2,610,185
Transposable elements content	57.35%
**Gene annotation statistics**	
Total number of protein-coding genes	24,015
Total exon number	136,156
Average exon number per gene	5.67
Average exon size (bp)	227.14
Total intro length (bp)	453,323
Total number of non-protein-coding genes	6,591

**FIGURE 2:**
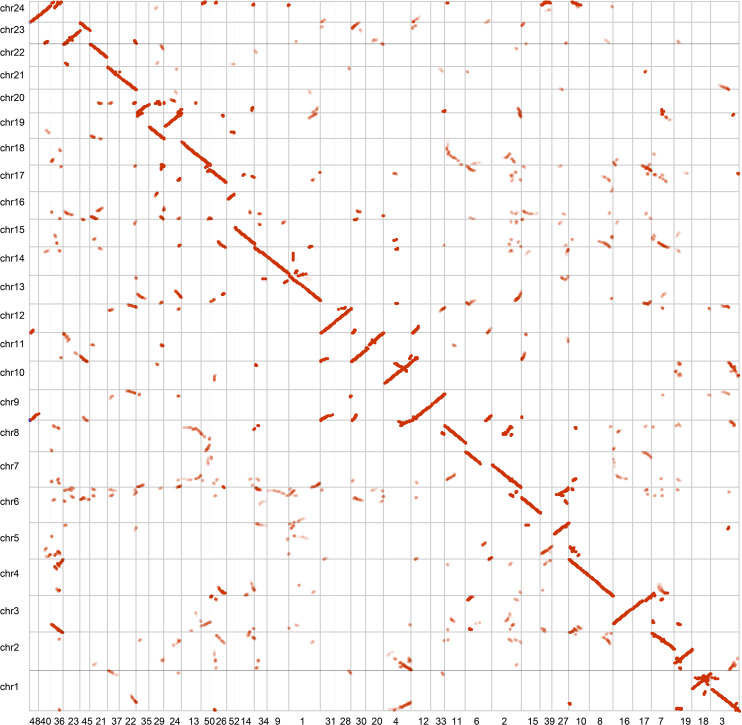
Collinear analysis of the assemblies of *A. paniculata* genome.

We assessed the completeness of the genome assembly of *A. paniculata* by using the Benchmarking Universal Single-Copy Orthologs (BUSCO) approach (Simao et al., 2015). The result showed that 91.0% of the plant BUSCO genes could be recovered in the genome assembly and 3% of the plant BUSCO genes had partial matches (Supplementary Table 2). We also mapped cleaned Illumina paired reads back to the genome assembly using BWA mem (Li and Durbin, 2010). We found that 97% of the reads could be mapped to the genome assembly and 94% of the reads were found to be properly paired. The high mapping rate indicate high completeness of the assembly. These two benchmarks were also higher than those reported in the previously assembly (Sun et al., 2019). In addition, our contig assembly shared high collinearity with its counterpart from a previous report (Figure 2). These data collectively suggest that the genome assembly of *A. paniculata* in this study has high quality and could be used for subsequent analyses.

**FIGURE 3:**
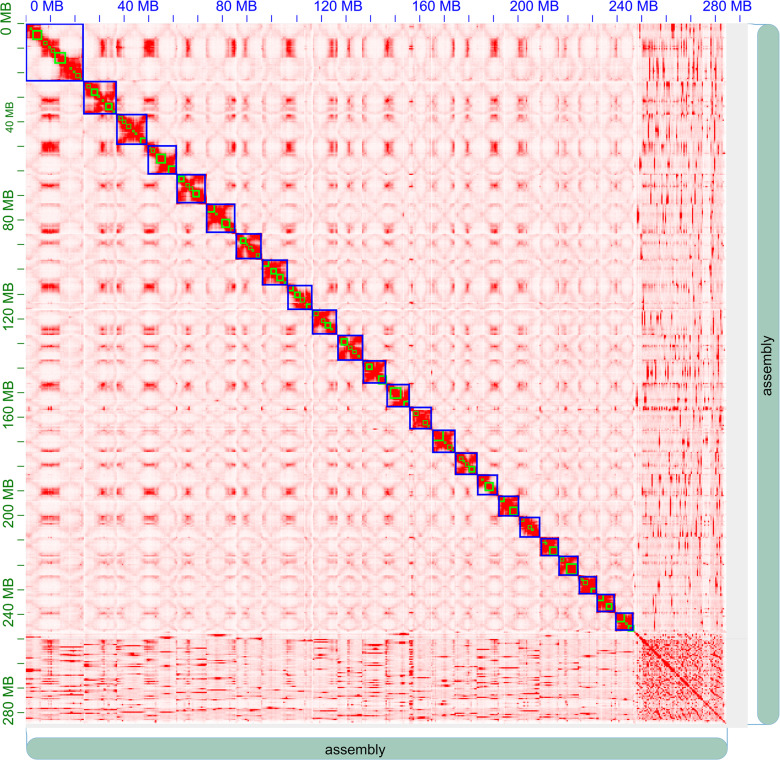
Hi-C clustering heatmap for pseudomolecule construction in *A. paniculata*. In all, 24 pseudomolecules are assembled and indicated by blue boxes.

Finally, we obtained about 43.33 Gb (∼152 × coverage) clean Hi-C sequencing data, with which 246,855,874 bp (86.8% of all bases) of the genome assembly were organized into 24 pseudo-chromosomes (Figure 3 and Supplementary Table 3). As expected, the Hi-C interaction decreases as the physical distance between two sequences increases.

### Repeat Annotation

**FIGURE 4:**
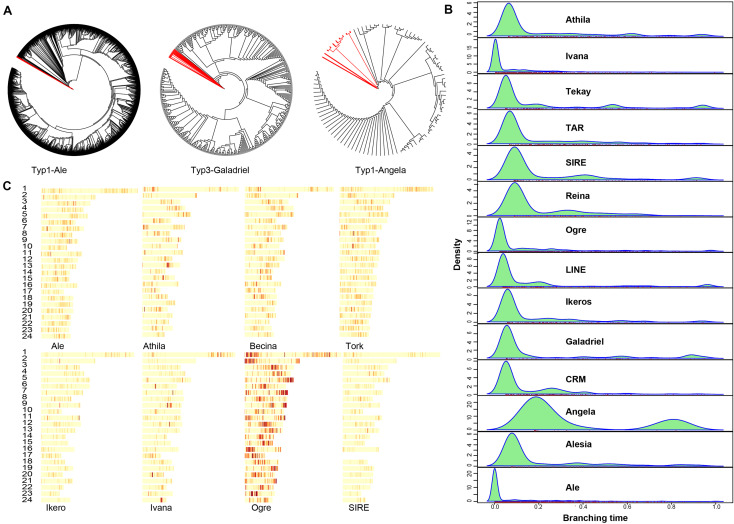
Repeat analysis **(A)** Neighbor-joining (NJ) trees were built from RT domain sequence similarities among different lineage-specific copies identified in the *A. paniculata* genome. Deep branching revealed ancient expansion while flat branching is consistent with a recent burst of insertion activity. Red branches correspond to outgroup sequences. **(B)** The average age of TEs was revealed for the different lineages by the branching distribution in the NJ trees built from RT (light blue). **(C)** The density of different TE lineages inferred from the detection of their protein-coding domains along pseudomolecules.

Transposable elements (TEs) accounted for about 163.1 Mb or 57.35% of the *A. paniculata* genome (Supplementary Table 4). Breakdown of the TE statistics showed that DNA retrotransposons and long terminal repeat (LTR) retrotransposons were major subtypes in the *A. paniculata* genome (Supplementary Table 5). TEs constitute an import part in plant genomes. We analyzed the evolutionary history of various *Ty3-gypsies* and *Ty1-copia* retroposon elements in the *A. paniculata* genome and identified unique dynamics of invasion patterns for different TE lineages (Figures 4A,B). For example, Ivana, Ogre, and Ale elements are all relatively young, suggesting an intense and recent burst of insertion or a strong selection against these TE elements. In contrast, Angela elements are the most ancient ones and the bimodal distribution suggests that the burst of insertion occurred twice in the evolutionary history of *A. paniculata* (Figure 4B). In addition, TE family distribution varied across the genome (Figure 4C). Ogre elements tend to cluster closely, thereby yielding prominent hotspot regions in the genome of *A. paniculata*.

### Protein-Coding Gene Annotation

**FIGURE 5:**
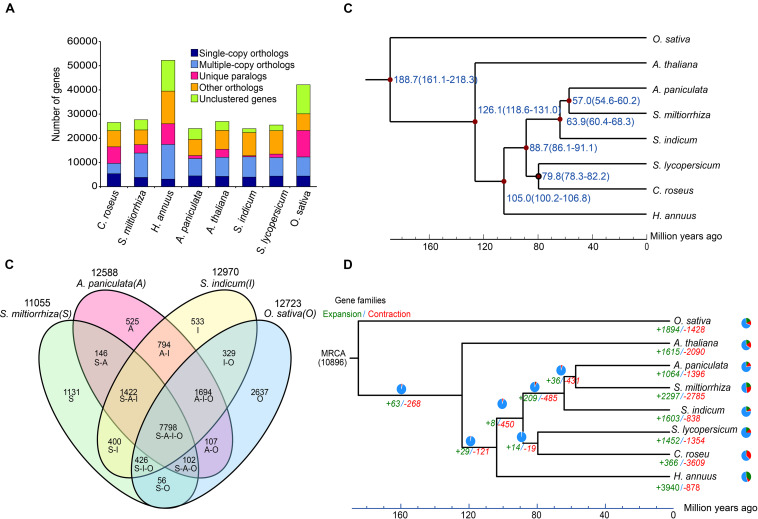
Comparative analyses of the *A. paniculata* genome. **(A)** Major groups of orthologous genes in eight plant genomes. **(B)** Venn diagram of shared orthologous gene families among *A. paniculata*, *S. indicum*, *S. miltiorrhiza*, and *O. sativa* genomes. **(C)** Estimation of the time points of divergence (time range shown in parentheses) between *A. paniculata* and seven other plant species based on orthologous single-copy gene pairs. **(D)** Expansion and contraction of gene families in eight plant genomes. Pie diagram on each branch and node corresponds to combined change across lineages.

A combination of *ab initio* based, homology based, and RNA-Seq based methods were used to predict 24,015 protein-coding genes in the *A. paniculata* genome (Supplementary Table 6). The predicted mRNA was on average 3,175 bp in length, containing about 5.67 exons with an average CDS length of 1,287 bp. The number of predicted protein-coding genes was comparable to that of *S. indicum*, but much smaller than that of *O. sativa* and *H. annuus* (Figure 5A). Orthologous clustering analysis showed that the *A. paniculata* genome contained 4,449 single-copy orthologs, 6,731 multiple-copy orthologs, 1,234 unique paralogs, 7,165 other orthologs, and 4,436 unclustered genes (Supplementary Table 7). In addition, Venn diagram showed that 7,798 gene families were shared by *A. paniculata*, *S. indicum, S. miltiorrhiza*, and *O. sativa*. A total of 525 gene families were unique to *A. paniculata*. This number was lower than that of the other three plant species (Figure 5B). Among the 24,015 protein-coding genes, a total of 19,824 predicted genes are supported by the RNA-seq expression data (FPKM > 0.05). Functional annotation of predicted protein-coding genes showed that 91.5% could obtain TrEMBL annotation; 62.2% could obtain GO annotation; 77.2% could obtain KEGG annotation; and 81.0% could obtain InterPro annotation (Supplementary Table 8).

### Non-protein-Coding Gene Annotation

A combination of homolog and *ab initio* based methods identified a total of 6,591 non-protein-coding genes (Supplementary Table 9). These predicted genes comprised of 93 microRNA genes, 524 transfer RNA (tRNA) genes, 5,785 ribosomal RNA (rRNA) genes, and 189 small nuclear RNA genes (Supplementary Table 9).

### Phylogenetic Analysis of *A. paniculata*

Phylogenetic analysis *A. paniculata* and seven other plant species showed that *A. paniculata* shared a common ancestor with *S. miltiorrhiza* approximately 57.0 Myr ago by calculated by r8s (Figure 5C). This estimate corresponds to the report from a previous study (Sun et al., 2019). During the course of evolution, a total of 1,064 and 1,396 gene families in *A. paniculata* were found to undergo expansion and contraction, respectively (Figure 5D).

### Genomic Analysis of Key Genes in the Terpenoid Biosynthetic Pathway

**FIGURE 6:**
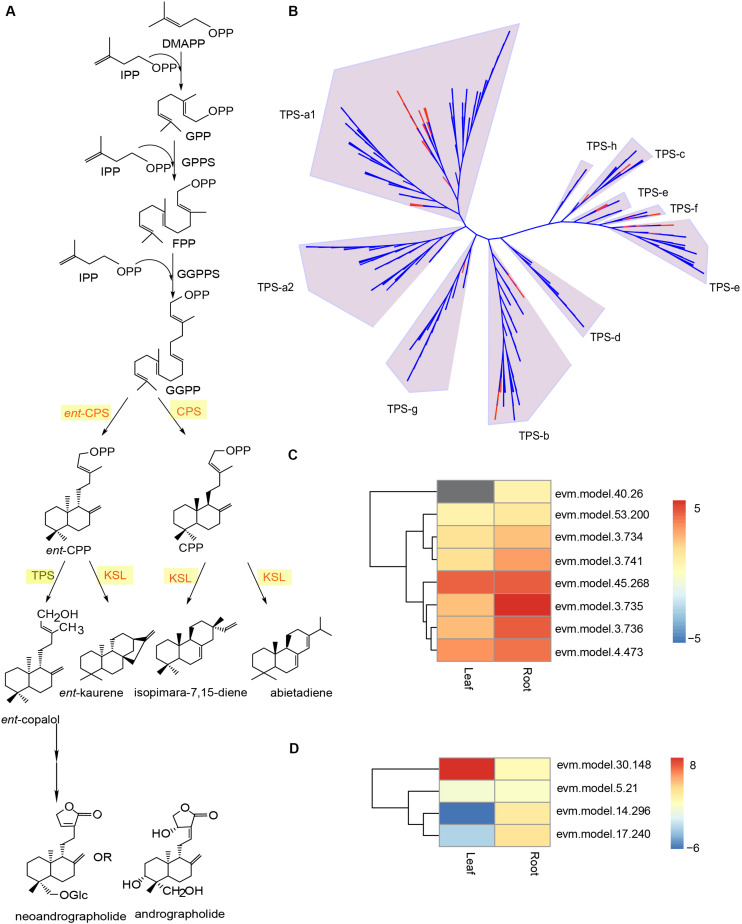
Genomic analysis of putative *TPS* genes in the *A. paniculata* genome. **(A)** Schematic biosynthetic pathway of andrographolide and neoandrographolide. **(B)** Phylogenetic analysis of *TPS* genes in *A. paniculata* and other plants. Red branches indicate putative *TPS* genes in *A. paniculata*. **(C)** Expression levels of putative *KS/KSL* genes in the leaf and root tissue of *A. paniculata*. **(D)** Expression levels of putative *CPS* genes in the leaf and root tissue of *A. paniculata*.

Terpenoids are the largest group of plant secondary metabolites that are key targets for pharmaceutical screening and design (Srivastava and Akhila, 2010). Despite the structural diversity, these compounds share a common biosynthetic pathway (Yazaki et al., 2017). Terpenoids are derived from two five-carbon chemicals: IPP and dimethylallyl diphosphate (DMAPP) (Lange et al., 2001). Their biosynthesis involve the classical acetate/mevalonate pathway in the cytosol and the pyruvate/glyceraldehyde-3-phosphate pathway in the plastids (Figure 6A) (Chen et al., 2011). Eventually, the condensation of IPP and DMAPP in various combinations will give rise to the countless terpenoids in plants (Srivastava and Akhila, 2010). In the *A. paniculata* genome, according to the KEGG annotation results, we identified 41 putative genes that were involved in the terpenoid backbone biosynthesis (Supplementary Table 10). This number is larger than that found in the *Panax notoginseng* genome (Chen et al., 2017). Additionally, almost all these putative genes exhibited certain levels of expression in the leaf and root tissue of *A. paniculata*. A previous report showed that the *GERANYLGERANYL PYROPHOSPHATE SYNTHASE* (*GGPPS*) group of genes in *A. paniculata* might be involved in the biosynthesis of andrographolide (Wang et al., 2019). In our genome assembly, a total number of 13 putative *GGPPS* genes were identified, and 11 out of 13 putative genes were expressed (Supplementary Table 10).

Besides *GGPPS* genes, previous studies suggest that *COPALYL DIPHOSPHATE SYNTHASE* (*CPS*) genes are implicated in the biosynthetic pathway of andrographolide and neoandrographolide (Garg et al., 2015; Shen et al., 2016a, b). The CPS enzymes are very similar in protein sequence to kaurene synthase (KS) and kaurene synthase-like (KSL) proteins (Zi et al., 2014). Despite their distinct catalytic activity, CPSs and KSLs belong to the c-type and e-type subfamilies of the terpene synthase (TPS) enzymes, respectively (Chen et al., 2011). In the *A. paniculata* genome, we identified a total of 53 putative *TPS* genes (Supplementary Table 11). Phylogenetic analysis of the TPS protein sequences from *A. paniculata* and eight other species showed that there were 24 putative *TPS-a1* genes, 10 putative *TPS-b* genes, 4 putative *TPS-c* genes, 8 putative *TPS-e* genes, 5 putative *TPS-f* genes, and 2 putative *TPS-g* genes (Figure 6B). This suggest that the *A. paniculata* genome may have four *CPS* genes and eight *KS*/*KSL* genes, all of which show certain levels of expression in the leaf and root tissues of the plant (Figures 6C,D).

### Genomic Analysis of the Cytochrome P450 Gene Family in *A. paniculata*

**FIGURE 7:**
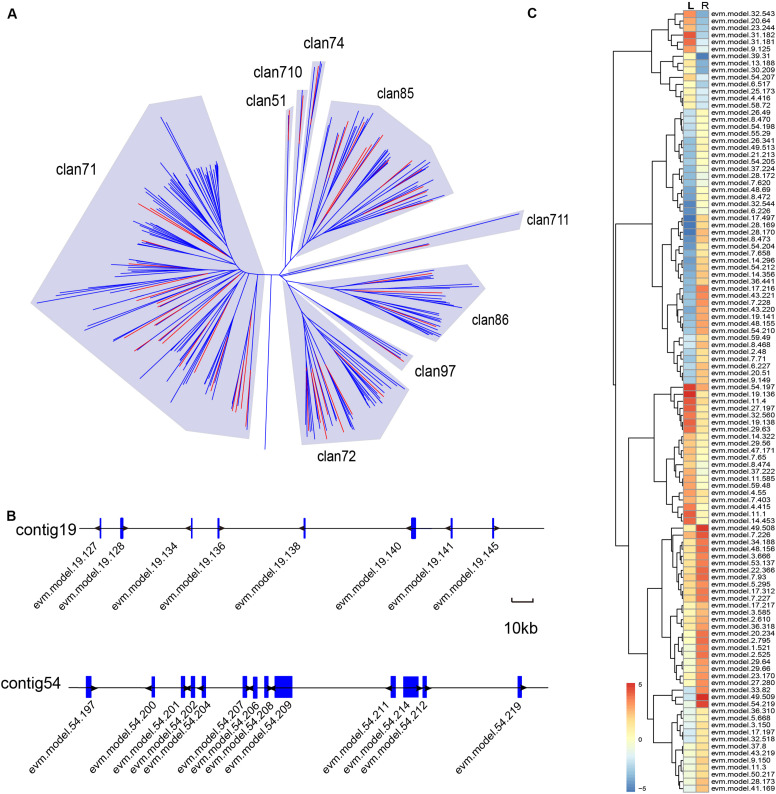
Genomic analysis of putative *CYP450* genes in the *A. paniculata* genome. **(A)** Phylogenetic analysis of *CYP450* genes in *A. paniculata* and other plants. Red branches indicate putative *CYP450* genes in *A. paniculata*. **(B)** The *CYP450* gene clusters on the contigs. **(C)** Differentially expressed putative *CYP450* genes in *A. paniculata*.

Various biosynthetic pathways in plants rely on members of the *CYTOCHROME P450* (*CYP450*) gene family to accomplish chemical modification (Schuler, 1996; Mizutani and Ohta, 1998; Morant et al., 2003). For this reason, we investigated the putative *CYP450* genes in *A. paniculata*, and found a total of 205 candidates (Supplementary Table 12). The phylogenetic tree of the putative CYP450 protein sequences exhibited nine major clans (Figure 7A). The majority of candidate genes belonged to the clan 71 (104 genes), clan 72 (33 genes), clan 85 (33 genes), and clan 86 (25 genes), respectively. We also found that some *CYP450* genes appeared in a cluster in the contigs of the assembly (Figure 7B). This result agrees with the findings that it is fairly common to have gene clusters for specific biosynthetic pathways in plant genomes (Nutzmann and Osbourn, 2014). Among the 205 candidate genes, we identified 111 putative *CYP450* genes that were differentially expressed in the root and leaf of *A. paniculata* (Figure 7C). Additionally, the expression level of evm.49.509 in the *CYP450* family was the highest in the root of *A. paniculata*, followed by evm.model.49.208, evm.model.7.226, and evm.model.54.219. In comparison, evm.model.19.136 was the highest expressed gene in the leaves of *A. paniculata*, followed by evm.model.54.197 and evm.model.11.4.

## Discussion

With the advancement and cost reduction of genome sequencing technologies, more and more plant species have revealed their near-complete genetic composition at a single-base resolution. Because most medicinal plant have highly repetitive and/or heterozygous genomes, a high-quality draft assembly is usually difficult and costly to secure. Particularly, lower contiguity of the genome assembly at the contig level will impede genomic analysis of functional genes, delineation of biosynthetic pathways, and the development of novel pharmaceutical candidate.

We reported a chromosome-level reference genome for *A. paniculata* with improved benchmark values. Our updated *A. paniculata* genome was 284 Mb with a contig N50 size of 5.14 M. This number was more than 12-fold longer than that of the previous report (Sun et al., 2019). The BUSCO value reached 91.7%, which demonstrated the high completeness of the genome assembly.

Repetitive elements account for a large percentage of plant genomes. For example, the genome of lettuce contains about 74.2% of the sequence as repeats and the genome of sunflower includes more than 75% LTRs (Badouin et al., 2017; Reyes-Chin-Wo et al., 2017). We analyzed the repetitive element sequences in the *A. paniculata* genome and found that the repeats of the *A. paniculata* genome is as high as 57%, among which long terminal repeat retrotransposons (LTR-RTs) are predominant. Phylogenetic analyses of various *copia* and *gypsies* RT subclasses showed that the burst of invasion of many RT were recent events except Angela elements. This is in contrast to the pea genome where Angela elements drives the invasion fairly recently (Kreplak et al., 2019). Given these results, it is worth noting that TE annotation remains a challenging task for plant genomes. For instance, about 21.8% of *de novo* identified TEs in the *A. paniculata* genome could not be assigned to a particular category. Moreover, TEs tend to insert into the structures of existing TE elements, creating nested TEs in the genome. With our data, it would be interesting to use newly developed pipelines (e.g., Extensitve *de novo* TE Annotator) (Ou et al., 2019) to deconvolute nested TEs in the genome of *A. paniculata.*

In the present study, we analyzed the *Cyp450* gene family in *A. paniculata*, and identified 205 putative *CYP450* genes with conserved motifs. The results showed that all major classes of *Cyp450* reported by the Nutzmann and Osbourn (2014) could be found in the *A. paniculata* genome. The number of *Cyp450* genes with high expressions is larger in roots than in leaf. Furthermore, terpene synthesis (TPSs) is one of the main drivers of terpene diversification. The terpene synthase (TPS) family genes are generally categorized into seven clades (Shen et al., 2018). In the *A. paniculata* genome, we identified a total of 53 putative *TPS* genes, most of which belong to the *TPS-a* and *TPS-b* subfamilies. This is in line with the fact that *TPS-a* and *TPS-b* subfamilies represent the angiosperm-specific genes that have diverged from the other *TPS* genes (Shen et al., 2018).

## Conclusion

The high-quality genome of *A. paniculata* will not only lay out the foundation for investigating the genetic basis for secondary metabolite biosynthesis, but also serve as an important resource for the study of other plant species in the *Andrographis* genus.

## Data Availability Statement

All raw sequence reads and the genome assembly have been deposited at NCBI under the BioProject accession number PRJNA549104.

## Author Contributions

YL, KW, and PQ collected the samples and performed the experiments. SC, ZY, YDu, and SD completed the data analysis. YDo, WC, and JM edited and modified the manuscript. All authors read and approved the manuscript.

## Conflict of Interest

The authors declare that the research was conducted in the absence of any commercial or financial relationships that could be construed as a potential conflict of interest.

## Footnotes

1 http://www.girinst.org/repbase/index.html

2 http://www.repeatmasker.org/

3 https://github.com/xzhub/LTR_Finder

4 http://www.drive5.com/piler/

5 http://www.repeatmasker.org/

6 http://www.repeatmasker.org/RepeatModeler.html

7 http://repeatexplorer.org/

8 https://itol.embl.de/

9 http://www.uniprot.org/

10 http://www.genome.jp/kegg/

11 https://www.ebi.ac.uk/interpro/

12 http://bioinfogp.cnb.csic.es/tools/venny/index.html

13 http://www.drive5.com/muscle

14 http://drnelson.uthsc.edu/CytochromeP450.html

## Abbreviations

BUSCO: Benchmarking Universal Single-Copy Orthologs
CPS: copalyl diphosphate synthase
CYP: cytochrome P450
DMAPP: dimethylallyl diphosphate
GGPPS: geranylgeranyl pyrophosphate synthase
GO: gene ontology
IPP: isopentenyl diphosphate
KSL: kaurene synthase-like protein
TE: transposable element.
